# Enhanced immunity: the gut microbiota changes in high-altitude Tibetan pigs compared to Yorkshire pigs

**DOI:** 10.3389/fmicb.2024.1469253

**Published:** 2024-11-18

**Authors:** Chengming Liu, Haifeng Dan, Yiting Yang, Yong Du, Ziling Hao, Lei Chen, Kangping Zhu, Bin Liu, Lili Niu, Ye Zhao, Yan Wang, Linyuan Shen, Mailin Gan, Li Zhu

**Affiliations:** ^1^State Key Laboratory of Swine and Poultry Breeding Industry, College of Animal Science and Technology, Sichuan Agricultural University, Chengdu, China; ^2^Key Laboratory of Livestock and Poultry Multi-omics, Ministry of Agriculture and Rural Affairs, College of Animal Science and Technology, Sichuan Agricultural University, Chengdu, China; ^3^Farm Animal Genetic Resources Exploration and Innovation Key Laboratory of Sichuan Province, Sichuan Agricultural University, Chengdu, China; ^4^Sichuan Dekon Livestock Foodstuff Group, Chengdu, China

**Keywords:** Tibetan pig, Yorkshire pig, gut microbiota, 16S rRNA, metagenome

## Abstract

**Introduction:**

Long-term domestication in high-altitude environments has led to unique changes in the gut microbiota of Tibetan Pigs. This study aims to investigate specific alterations in the intestinal flora of Tibetan Pigs compared to Yorkshire pigs.

**Methods:**

We employed 16S rRNA and metagenomic sequencing technologies for comprehensive analysis of the gut microbiota. The data collected allowed us to assess microbial community structures and functional capabilities.

**Results:**

Our analysis revealed that Tibetan Pigs raised under a “free-range + supplementary feeding” model exhibited increased abundance of microbial communities associated with short-chain fatty acid synthesis and the digestion of cellulose and hemicellulose. Notably, the characteristic bacterium *Rhodococcus*, commonly found in high-altitude environments, was enriched in the gut microbiota of Tibetan Pigs, facilitating the efficient utilization of natural compounds and degradation of toxic substances. Additionally, the increased abundance of probiotics in these pigs enhances their immunity, which may involve mechanisms such as disrupting the structure of pathogenic bacteria and detoxifying harmful metabolites.

**Discussion:**

These findings underscore the advantages of Tibetan Pigs over common commercial breeds, highlighting their unique gut microbiota adaptations. Furthermore, they open new avenues for screening potential probiotics and developing genetic breeding strategies for improved livestock varieties.

**Conclusion:**

Understanding the distinct gut microbiota of Tibetan Pigs provides valuable insights into their health benefits and resilience, contributing to future research on breed improvement and microbiome applications in agriculture.

## 1 Introduction

The Tibet pig is a unique indigenous fatty-type breed in China, primarily found in the Tibet Autonomous Region, Sichuan Province, Yunnan Province, and Gansu Province. It is characterized by strong fat deposition capability, disease resistance, stress tolerance (Shang et al., 2022), adaptation to low-oxygen conditions (Ma et al., 2019; Yang et al., 2022), and resilience to roughage. The Yorkshire pig is a classic lean meat breed and ranks among the most widely raised pig breeds worldwide. It is celebrated for its rapid weight gain, efficient feed conversion, and notable yield of lean meat. In contrast to the large-scale intensive farming methods used for Yorkshire pigs, Tibetan pigs are typically raised using a blend of high-altitude grazing and confinement. This allows them to consume more high-fiber foods during feeding. The distinct living environment and husbandry practices contribute to Tibetan pigs’ enhanced disease resistance while also showcasing excellent fat deposition characteristics (Niu et al., 2022).

As a vital part of the gut microbiota, intestinal bacteria profoundly influence the host’s health and physiological functions. Primarily, they impact the host’s metabolism. In the human body, dietary fibers such as lignin, non-starch polysaccharides, resistant starch (RS), and oligosaccharides resist digestion by host enzymes, thereby impeding normal absorption and utilization (Anderson et al., 2009). However, gut microbiota possess a variety of enzymes that metabolize these diverse carbohydrates, breaking them down into short-chain fatty acids and small amounts of organic acids for absorption and utilization by the human body (Louis and Flint, 2009). Undigested proteins can also be degraded by extracellular bacterial proteases and peptidases into peptides, amino acids, and other metabolites. This process plays a crucial role in regulating the gut–brain axis and maintaining the host’s nitrogen balance (Adak and Khan, 2019). Furthermore, the gut microbiota regulates the immune system by interacting with immune cells in extraintestinal organs through various mechanisms. Gut microbiota directly influences immune cell function and activity by adhering to cell surfaces or being engulfed by phagocytic cells (Iimura et al., 2005). They can also activate immune cells by binding to receptors on the surfaces of intestinal epithelial cells and macrophages, thereby triggering immune responses and pro-inflammatory signals. This interaction further regulates the activity of immune cells (Ivanov et al., 2008). At the same time, the gut microbiota communicates with immune cells by producing metabolites such as short-chain fatty acids and other microbial molecules. These metabolites directly regulate the activity and function of immune cells, impacting their proliferation, differentiation, and production of effector molecules (Hubbard et al., 2015; Morrison and Preston, 2016). The gut microbiota is intricately linked to the onset of diseases. For instance, a decrease in bacteria belonging to the phylum Bacteroidetes and an increase in Firmicutes and Proteobacteria can result in excessive fat accumulation, thereby contributing to obesity (Torres-Fuentes et al., 2017; Duan et al., 2021). In addition, certain metabolites produced by the gut microbiota have been demonstrated to induce colorectal cancer and renal dysfunction (Iatcu et al., 2021; Bai et al., 2022).

Through adaptation to high-altitude environments, the gut microbiota of Tibetan pigs have undergone distinctive alterations. Research indicates that Tibetan pigs, compared to those raised at low altitudes, show a significantly higher abundance of Fibrobacteres in the gut, along with increased levels of carbohydrate utilization genes and *α*-diversity indices (Zeng et al., 2020). Building on previous studies, this research aimed to delve deeper into the distinct changes in composition and functional dynamics of the gut microbiota in Tibetan pigs.

## 2 Materials and methods

### 2.1 Sample and trait data collection

Fecal samples were collected from Tibetan pigs in Xiangcheng County, Ganzi Tibetan Autonomous Prefecture, Sichuan Province, and Yorkshire pigs in Fushun County, Zigong City, Sichuan Province. A total of six Tibetan pigs and six Yorkshire pigs were slaughtered for sampling. The Tibetan pigs were sourced from local farmers, while the Yorkshire pigs were selected from a breeding farm, ensuring that all animals were sexually mature and met the slaughter weight standards. Prior to slaughter, all pigs had free access to water and feed but were fasted for 24 h while still allowed to drink water. Fecal samples were collected within 10 min post-slaughter and immediately placed on dry ice before being transferred to a − 80°C freezer for storage.

### 2.2 16S rRNA and metagenomic sequencing of fecal microbiota DNA

The collected fecal samples were sent to Beijing NovogeneAIT Genomics Technology Co., Ltd. for total DNA extraction, DNA quality assessment, and library preparation for sequencing. Each fecal sample underwent sequencing of the 16S rRNA gene V3-V4 region and metagenomic sequencing. The Illumina NovaSeq platform (PE250) was used for 16S rRNA sequencing, while the Illumina platform was used for metagenomic sequencing (PE150).

### 2.3 Bioinformatic analysis of fecal 16S rRNA sequencing data

The 16S rRNA data were analyzed using Qiime2 (v2022.8.3) software and its integrated plugins. Initially, the qiime tools import plugin was used to import single-end 16S rRNA sequencing data. Subsequently, the qiime dada2 denoise-paired plugin and qiime feature-table filter-features plugin were used to denoise and filter the raw data. The filtered data were then used to construct a phylogenetic tree using the qiime phylogeny align-to-tree-mafft-fasttree plugin. For *α*-diversity and *β*-diversity analyses, the qiime diversity core-metrics plugin was utilized. Species annotation was performed using the qiime feature-classifier plugin. Finally, functional prediction of OTU abundance tables was carried out using PICRUSt2 (v2.5.2).

### 2.4 Bioinformatic analysis of fecal metagenomic sequencing data

Raw data obtained from the Illumina sequencing platform were processed using Trimmomatic (v0.39) software to obtain clean data. Subsequently, Bowtie2 software was used to align the clean data with the pig reference genome sequence (Sus11.1) to filter out reads possibly originating from the host (Karlsson et al., 2012, 2013; Scher et al., 2013). The clean data obtained from quality control were assembled into scaftigs using Megahit (v1.2.9) software (−-presets meta-large) for individual samples, removing host sequences, and filtering out fragments less than 500 bp in length (Li et al., 2014; Zeller et al., 2014; Sunagawa et al., 2015). These assemblies were used for subsequent gene prediction. MetaGeneMark (v3.42) software was used to predict open reading frames (ORFs) from the assembly results of each sample, filtering out results shorter than 100 bp. Subsequently, CD-HIT (v4.8.1) software was used to construct non-redundant gene sets (parameters: -c 0.95, −G 0, -aS 0.9, −g 1, −d 0). Finally, Bowtie2 (v2.5.1) was used to map clean data to the non-redundant gene sets to obtain reads count per gene in each sample, filtering out genes with reads count ≤2. The remaining unigenes were used for subsequent analyses (Oh et al., 2014; Qin et al., 2014). DIAMOND (v2.1.8) software was used to align unigenes with sequences extracted from the NCBI NR (Version: 2023.03) database, encompassing bacteria, fungi, archaea, and viruses, to obtain taxonomic annotation information. Unigenes were also compared with the KEGG database for functional annotation information. In cases where a unigene had multiple annotation results, alignments with an E-value ≤ Min E-value * 10 were selected for further analysis. In the LEfSe analysis for identifying differential bacterial communities, a threshold of |LDA score| > 2 and *p*-value <0.05 was chosen to broadly identify significant differences in bacterial populations (Qi-Xiang et al., 2022; Yu et al., 2022).

## 3 Results

### 3.1 Overview of trait statistics and data quality

After completing data preprocessing steps, including quality control, noise reduction, and alignment, over 90% of the metagenomic data remained available for subsequent analysis, averaging 99.71%. For the 16S rRNA data, following quality control and denoising, effective data retention rates ranged from 84.08 to 90.15% across all samples, with a mean of 87.76% (Supplementary Table S1). Furthermore, according to measurements of body weight and dimensions, adult Tibetan pigs demonstrate significantly lower body weight and smaller physique compared to Yorkshire pigs, highlighting a considerable contrast between these two breeds (Table 1).

**Table 1:** Statistical analysis and *t-*test results of weight and body size in Tibetan and Yorkshire pigs.

Traits	Breed		*P*-value
	Tibetan pig	Yorkshire pig	
Weight (kg)	37.5 ± 1.44	102.33 ± 7.94	<0.0001
Body length (cm)	73.0 ± 1.26	108.67 ± 5.32	0.0022
Head length (cm)	20.58 ± 1.02	24.83 ± 1.33	<0.0001
Chest circumference (cm)	65.17 ± 1.94	99.67 ± 2.79	<0.0001
Chest width (cm)	16.5 ± 1.61	27.75 ± 1.41	0.0022
Chest depth (cm)	25.17 ± 1.72	34.83 ± 2.14	<0.0001
Abdominal circumference (cm)	75.83 ± 4.96	103.67 ± 2.18	<0.0001
Hip circumference (cm)	67.5 ± 6.19	117 ± 7.51	<0.0001
Shank circumference (cm)	11.58 ± 0.8	17.25 ± 0.76	0.0022

### 3.2 Significant differences in gut microbiota diversity between Tibetan pigs and Yorkshire pigs

**Figure 1:**
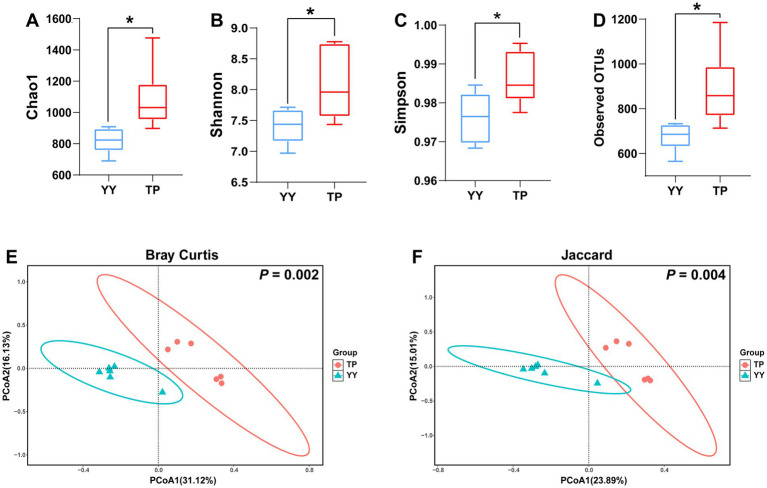
*α*- and-*β* diversity analysis results of 16S rRNA data between Tibetan Pigs and Yorkshire pigs: **(A–D)** *α*-diversity statistics of 16S rRNA data between Tibetan pigs and Yorkshire pigs, including Chao1 index, Shannon index, Simpson index, and number of observed OTUs and (E and F) *β* diversity PCoA results of 16S rRNA data between Tibetan Pigs and Yorkshire pigs, represented by PCoA plots based on Bray–Curtis distance and Jaccard distance.

Based on 16S rRNA sequencing data, we analyzed the species diversity between two populations. The *α*-diversity results revealed that the Chao1 index, Shannon index, Simpson index, and observed OTUs of gut microbiota in Tibetan pigs were significantly higher than those in Yorkshire pigs (Figures 1A–D). *β*-diversity analysis using Bray–Curtis and Jaccard distances indicated distinct clustering of Tibetan pigs and Yorkshire pigs in the PCoA plot within the 99% confidence interval (Figures 1E,F). The diversity analysis of the 16S rRNA data demonstrated significant differences in species diversity and structural composition of intestinal flora between Tibetan pigs and Yorkshire pigs, with Tibetan pigs showing significantly higher species diversity in their intestinal flora.

### 3.3 Comparative analysis of gut microbiota structure and function between Tibetan pigs and Yorkshire pigs

**Figure 2:**
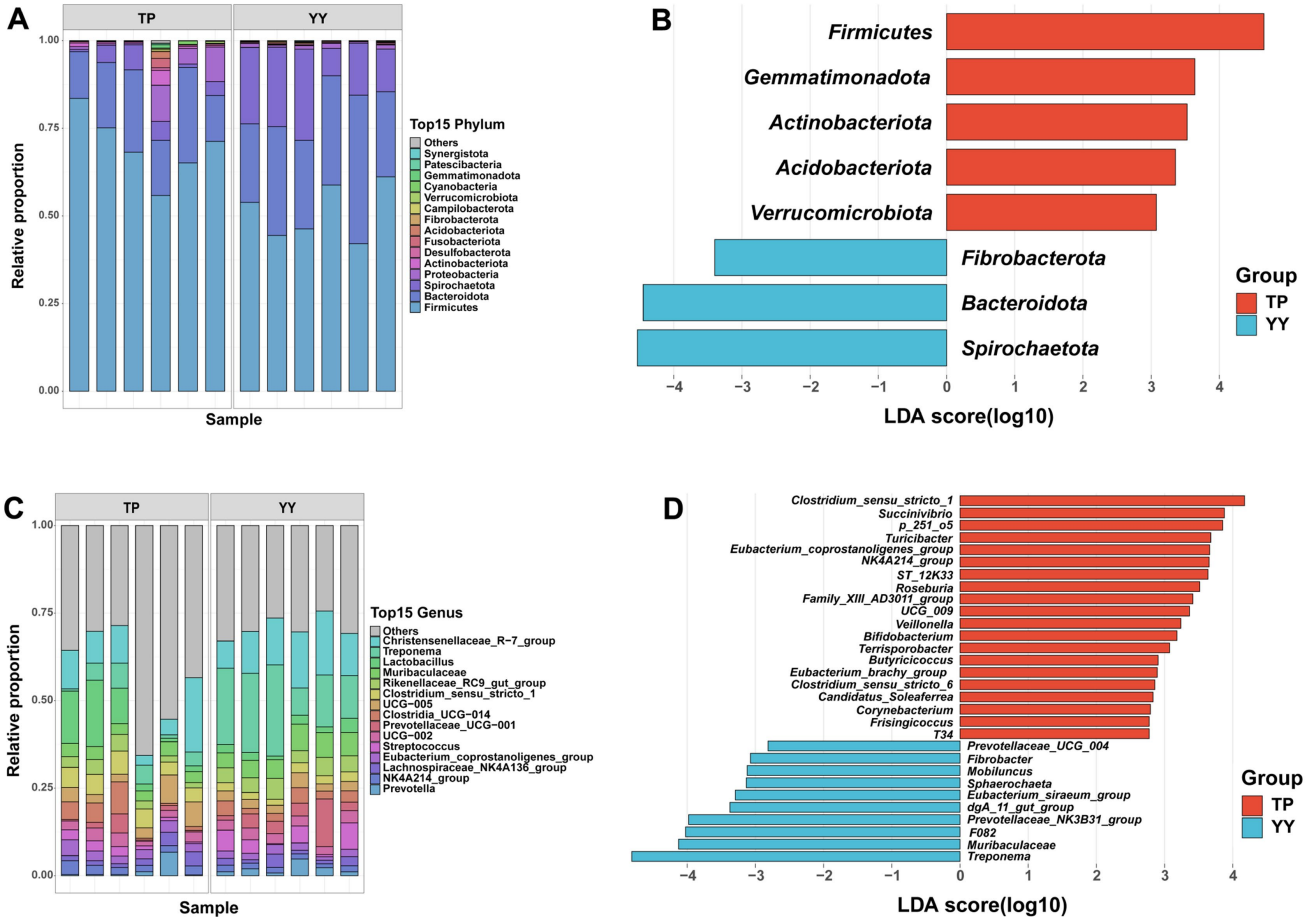
Based on 16S rRNA data species annotation results: **(A,B)** species annotation and LEfSe analysis results of phylum level; **(C,D)** species annotation and LEfSe analysis results of genus level.

Based on 16S rRNA data, species annotation and functional prediction analyses of OTUs obtained from Tibetan pigs and Yorkshire pigs were conducted. The species annotation results revealed that at the phylum level, Firmicutes (70.41% vs. 51.28%), Bacteroidota (18.68% vs. 29.09%), and Spirochaetota (3.75% vs. 17.67%) were the three predominant gut microbiota in Tibetan pigs and Yorkshire pigs (Figure 2A). At the genus level, the top three genera in Tibetan pigs and Yorkshire pigs were Christensenellaceae_R-7_group (10.22% vs. 13.11%), Treponema (3.75% vs. 17.66%), and Lactobacillus (8.32% vs. 2.34%) (Figure 2C). After LEfSe analysis (*p* < 0.05, |LDA| > 2), a total of 8 differential phyla (Figure 2B) and 53 differential genera (Figure 2D) were identified between the two populations. Tibetan pigs exhibited an increased abundance of Firmicutes and a decreased abundance of Bacteroidota and Spirochaetota at the phylum level. At the genus level, further analysis revealed significant reductions in Prevotellaceae, which plays a role in intestinal flora homeostasis (Zhou et al., 2021; Sánchez-Pérez et al., 2022), in Tibetan pigs. Conversely, genera such as *Dorea*, *Eubacterium*, *Butyricicoccus*, and *Lachnospiraceae*, known as potential intestinal probiotics, were significantly enriched in Tibetan pigs (Geirnaert et al., 2014; Mukherjee et al., 2020; Vacca et al., 2020; Xu et al., 2021). In addition, *Rhodococcus* (Kim et al., 2018; Alvarez et al., 2021), a genus characteristic of high-altitude environments, was also found to be significantly more abundant in Tibetan pigs.

**Figure 3:**
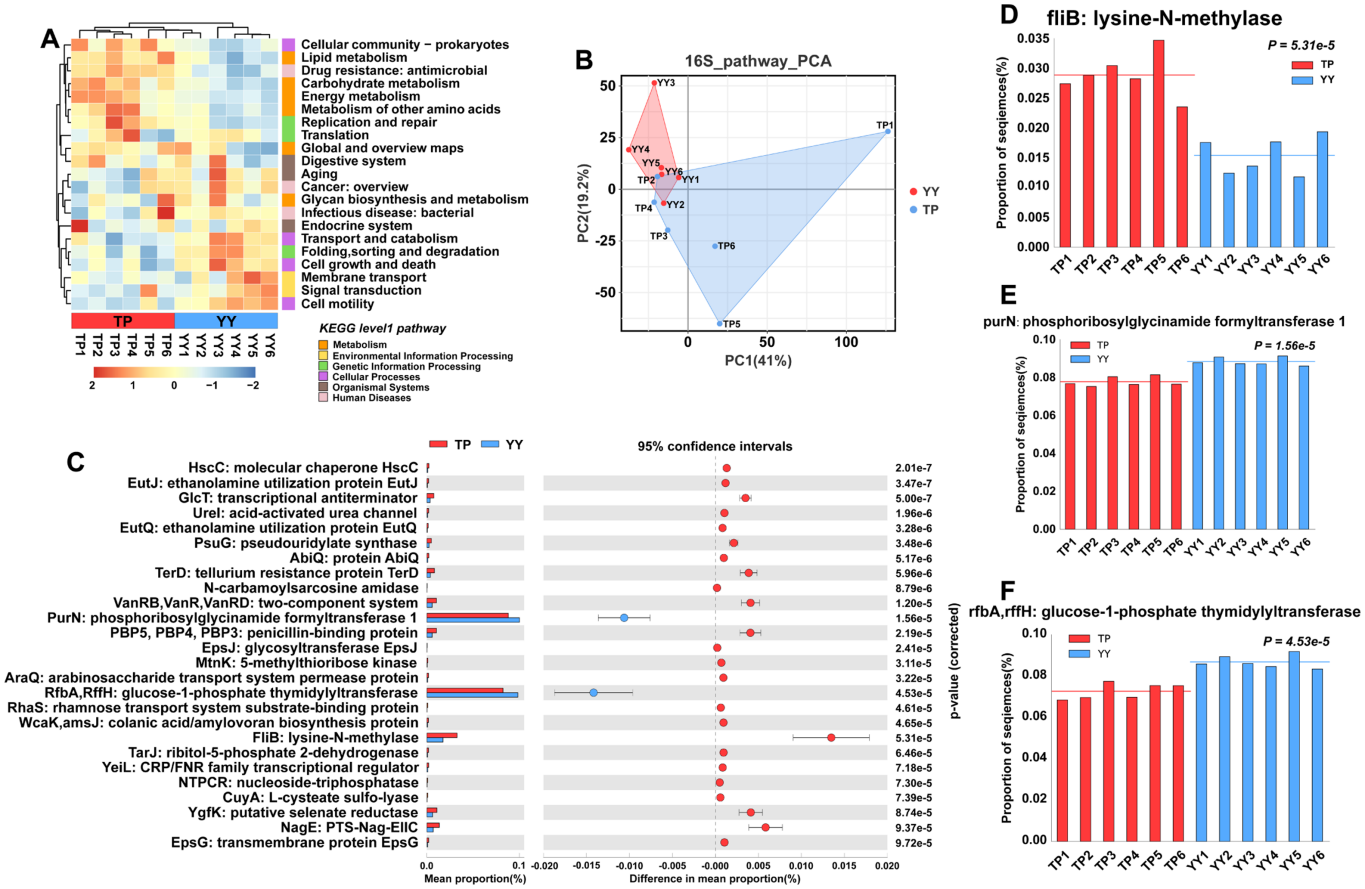
Functional prediction based on 16S rRNA data: **(A)** KEGG pathway heatmaps at level 1 and level 2; **(B)** PCA results of functional pathway prediction for Tibetan pigs and Yorkshire pigs; **(C)** differential pathways between Tibetan pigs and Yorkshire pigs; and **(D–F)** grouped bar chart of top 3 differential pathways between Tibetan pigs and large white pigs.

In the results of functional prediction using the KEGG database at level 1 and level 2 pathways, significant differences between Tibetan pigs and Yorkshire pigs were predominantly observed in metabolic pathways, including carbohydrate, lipid, and amino acid metabolism (Figure 3A). Furthermore, principal component analysis (PCA) indicated that the functional distinctions between Tibetan pigs and Yorkshire pigs were not pronounced, likely due to substantial intra-group variations among Tibetan pigs (Figure 3B). Based on differential analysis of EC-level pathways in the KEGG database, a total of 26 pathways showed significant differences (Figure 3C). The three pathways with the most pronounced distinctions were fliB: lysine-N-methylase pathway, purN: phosphoribosylglycinamide formyltransferase 1 pathway, and rfbA,rffH: glucose-1-phosphate thymidylyltransferase pathway (Figures 3D–F). Among them, fliB, influencing Salmonella colonization in the host’s intestinal tract (Horstmann et al., 2020), and purN, associated with *Staphylococcus aureus* antibiotic resistance (Peng et al., 2022), were found to be enriched in Tibetan pigs. In contrast, rfbA/rffH, involved in antibacterial compound synthesis (Sepúlveda-Correa et al., 2021), exhibited enrichment in Yorkshire pigs.

In summary, despite Tibetan pigs displaying a higher abundance of probiotics in their intestines, which aids in maintaining intestinal stability, functional prediction results indicate that these alterations in the gut microflora might not effectively combat environmental bacterial invasions.

### 3.4 Exploring gut microbiome differences between Tibetan pigs and Yorkshire pigs based on metagenomic analysis

**Figure 4:**
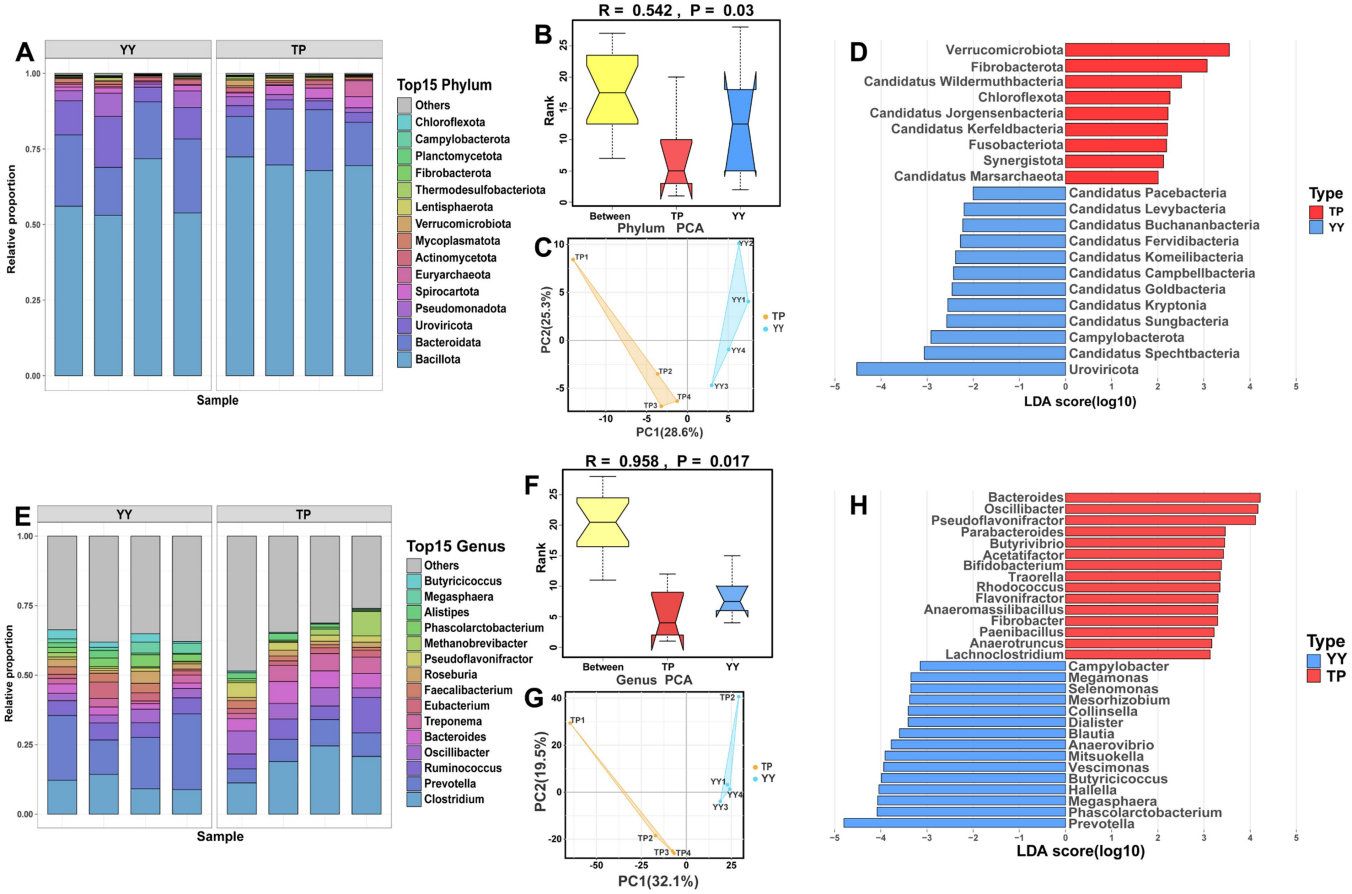
Species annotation at the phylum and genus levels and differential microbial community analysis between Tibetan pigs and Yorkshire pigs: The stacked species distribution diagram at phylum **(A–D)** and genus **(E–H)** levels, ANOSIM results, PCA results, and LEfSe analysis results for Tibetan pigs and Yorkshire pigs.

Based on metagenomic sequencing data, unigenes were constructed through quality control, assembly, and redundancy removal for subsequent analysis. A total of 175 annotated phyla were identified from the unigene. The dominant phyla in the gut microbiota of Tibetan pigs and Yorkshire pigs were Firmicutes (70.02% vs. 58.70%), Bacteroidetes (16.30% vs. 20.72%), and Proteobacteria (3.23% vs. 10.79%). Analysis of similarity (ANOSIM) and PCA further underscored significant differences in gut microbiota composition between Tibetan pigs and Yorkshire pigs (Figures 4A–C). LEfSe analysis revealed notable distinctions in the gut microbiota profiles of the two breeds (Figures 4D). Tibetan pigs exhibited significantly higher abundances of 9 phyla including Actinobacteria and Fibrobacteres, while Yorkshire pigs showed significantly higher abundances of 12 phyla including Spirochaetes and Bacteroidetes.

At the genus level, *Clostridium* (18.61% vs. 16.22%) was found to be predominant in both Tibetan pigs and Yorkshire pigs, followed by Prevotella (7.80% vs. 8.32%) and *Ruminococcus* (7.66% vs. 3.10%). ANOSIM and PCA confirmed significant differences in gut microbiota composition between Tibet pig and Yorkshire pig populations at the genus level (Figures 4E–G). LEfSe analysis highlighted specific differences in genus abundance between the Tibet pig and Yorkshire pig (Figure 4H). Tibetan pigs exhibited a significantly higher abundance of 172 genera including *Bacteroides*, *Spirochaeta*, and *Pseudoflavonifractor*. In contrast, Yorkshire pigs showed a significantly higher abundance of 30 genera including Prevotella, Phascolarctobacterium, and Megamonas than Tibetan pigs.

Overall, these findings highlight substantial differences in gut microbiota composition between Tibetan pigs and Yorkshire pigs, emphasizing the role of the breed in shaping microbial communities within the gut.

### 3.5 Consistency in functional predictions between metagenomics and 16S rRNA analysis

**Figure 5:**
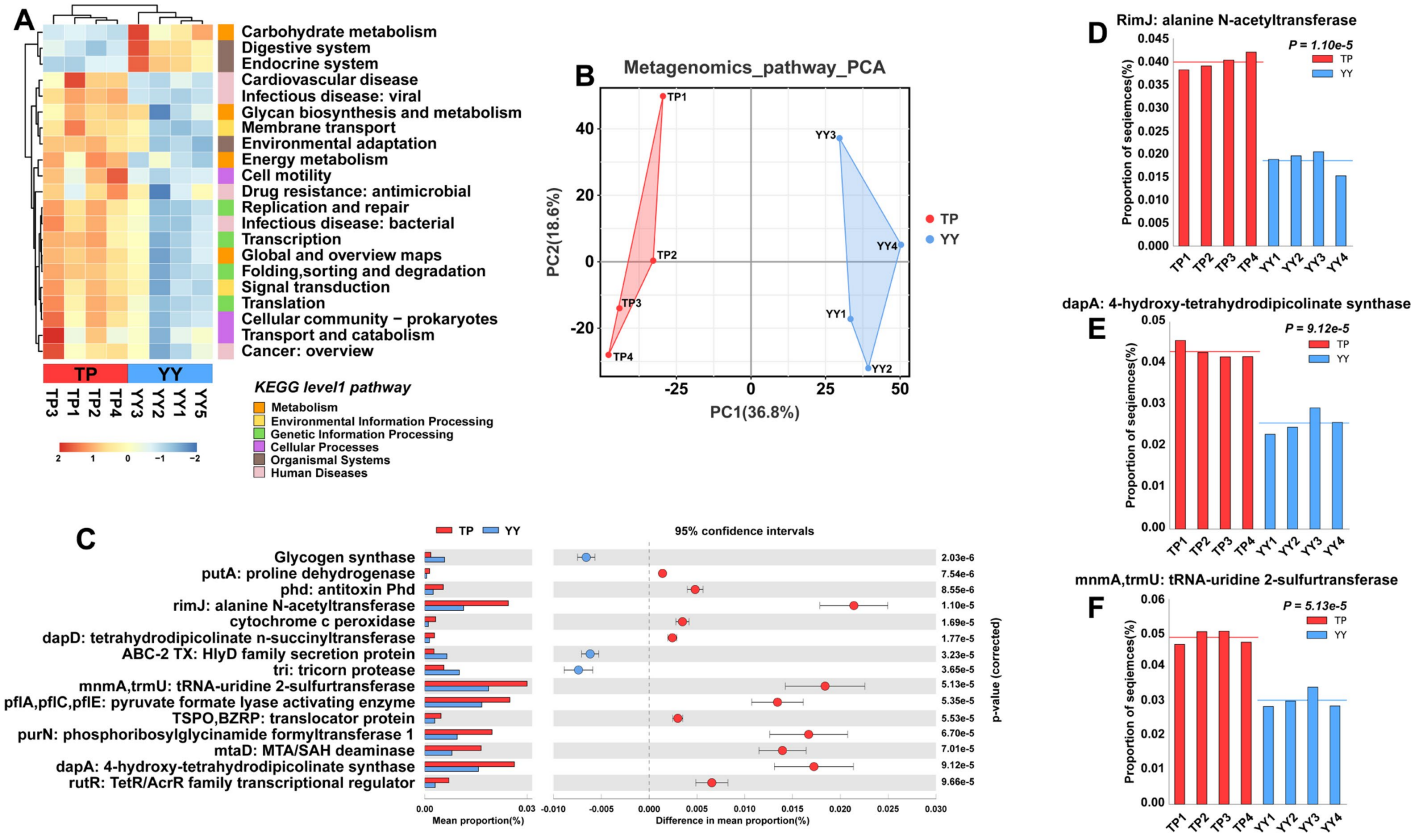
Functional prediction based on metagenome data: **(A)** KEGG pathway heatmaps at level 1 and level 2; **(B)** PCA results of functional pathway prediction for Tibetan pigs and Yorkshire pigs; **(C)** differential pathways between Tibetan pigs and Yorkshire pigs; and **(D–F)** grouped bar chart of top 3 differential pathways between Tibetan pigs and large white pigs.

Similar to predictions based on 16S rRNA analysis, Tibetan pigs and Yorkshire pigs show notable differences in level 1 and level 2 pathways according to metagenomic analysis (Figures 5A,B). However, in contrast to the 16S rRNA predictions, the functional disparities identified through metagenomic analysis primarily center around pathways related to disease, such as cardiovascular disease, Drug resistance: antimicrobial pathways, the second is the metabolism pathway. It is noteworthy that the metabolic-related pathways predicted by metagenomic functional analysis align closely with the predictions from 16S rRNA analysis. These pathways include carbohydrate metabolism, energy metabolism, global and overview maps, and pathways related to glycan biosynthesis and metabolism. Further analysis of EC-level pathways revealed 15 pathways that differed significantly between Tibetan pigs and Yorkshire pigs (Figure 5C). The three pathways showing the most pronounced differences were the RimJ: alanine N-acetyltransferase pathway, MnmA,TrmU: tRNA-uridine 2-sulfurtransferase pathway, and dapA: 4-hydroxy-tetrahydrodipicolinate synthase pathway (Figures 5D–F). All three pathways were notably enriched in Tibetan pigs. Specifically, rimJ inhibits transcription in response to environmental stimuli in *E. coli* (White-Ziegler et al., 2002), mnmA and trmU are essential for synthesizing the broad-spectrum antiviral nucleoside analog 2-thiouridine (Kambampati and Lauhon, 2003), while dapA regulates bacterial adhesion (Soo et al., 2005). Similar to the functional predictions derived from 16S rRNA analysis, the metagenomic functional predictions also indicate that the gut microbiota of Tibetan pigs play a significant role in maintaining intestinal stability. However, they may exhibit deficiencies in functions related to resisting bacterial invasion and colonization.

## 4 Discussion

As a unique local breed in China, Tibetan pigs exhibit excellent tolerance to coarse feed, along with superior capability for fat deposition compared to Yorkshire pigs, and stronger resilience to adversity. The gut microbiota composition in Tibetan pigs differs significantly from that in Yorkshire pigs, reflecting their distinct *α* and *β* diversity patterns. Tibetan pigs exhibit significantly higher α diversity, which aligns with previous research indicating the impact of their feeding practices on microbiota diversity and immune enhancement (Zeng et al., 2020). The 16S rRNA species annotation results indicated that Tibetan pigs exhibit an increased abundance of Firmicutes and a decreased abundance of Bacteroidetes and Spirochaetes. Studies on Min pigs (Zhao et al., 2022) suggested that decreased Firmicutes and Bacteroidetes, along with Spirochaetes, are characteristic of intestinal flora in pigs with colon cancer. These microbial changes are closely associated with inflammatory responses in the pig intestine. In Tibetan pigs, these alterations potentially contribute to maintaining intestinal stability and reducing inflammation occurrence. The characteristic genus *Rhodococcus* (Kim et al., 2018; Alvarez et al., 2021) is notably enriched in Tibetan pigs inhabiting high-altitude environments. These bacteria possess the ability to decompose and transform various natural compounds through diverse metabolic pathways. Moreover, they exhibit tolerance to toxic substrates and solvents, aiding Tibetan pigs in adapting to the challenging conditions of high altitudes. However, the functional prediction results indicate significant changes. In addition to an increase in pathways affecting Salmonella toxicity in Tibetan pigs, there is a decrease in pathways associated with *Staphylococcus aureus* resistance and antibacterial compound synthesis. Overall, these differential pathway results suggest that Tibetan pigs may be more susceptible to Salmonella, with diminished capacity to synthesize antibacterial substances. Considering the robust resistance of Tibetan pigs, it is hypothesized that their complex intestinal flora plays a crucial role in detoxification. This flora likely maintains homeostasis in the intestinal environment by either disrupting the integrity of the bacterial structure or degrading toxic compounds. Changes in the abundance of highly metabolically and degradative-capable bacteria, such as *Rhodococcus*, further support these speculations.

Previous studies have demonstrated that supplementing roughage in the diet enhances the populations of fiber-degrading bacteria and increases the production of short-chain fatty acids in Tibetan pigs. This dietary intervention plays a crucial role in strengthening their immune defenses (Gao et al., 2022). Metagenomic sequencing data analysis highlights significant differences in microbial genus between the two pig populations. Notably, the abundance of Oscillospira in Tibetan pigs is markedly higher, known for its role in maintaining immune stability across various growth stages (Zafar and Saier Jr, 2021). Other genera such as *Pseudoflavonifractor*, *Parabacteroides*, and *Butyrivibrio*, also identified with increased abundance in Tibetan pigs, contribute to short-chain fatty acid production essential for gut health and metabolic functions. Among these genera, *Pseudoflavonifractor* contributes to the production of short-chain fatty acids to maintain intestinal homeostasis (Borda-Molina et al., 2016). *Parabacteroides*, in addition to its physiological characteristics in carbohydrate metabolism and secretion of short-chain fatty acids (Cui et al., 2022), also plays a role in alleviating obesity and metabolic disorders (Wang et al., 2019). *Butyrivibrio* is one of the most common bacteria in the rumen microbiota of ruminants but is also found in the gastrogut microbiota of mammals (Rodríguez Hernáez et al., 2018). Its primary function involves the degradation of plant polysaccharides and the fermentation of released monosaccharides to produce short-chain fatty acids (Palevich et al., 2019; Zhu et al., 2022). The high abundance of *Rhodococcus* (Kim et al., 2018; Li et al., 2018) in Tibetan pigs suggests that they possess enhanced metabolic detoxification abilities, which help maintain intestinal homeostasis. On the other hand, *Campylobacter*, known as a pathogen causing intestinal diseases such as enteritis and diarrhea, shows a significantly reduced presence in Tibetan pigs, indicating a lower risk of diarrhea in this population (Moore et al., 2005). In the functional prediction results of the metagenome, consistent findings were observed compared to the 16S rRNA functional predictions. There was an increase in pathways associated with bacterial invasion and colonization within the Tibet pig population, while certain pathways linked to antibacterial or antiviral synthesis showed decreased abundance. In summary, the exceptional disease resistance of Tibetan pigs is attributed to their robust immunity. However, prolonged exposure to the natural environment while being fed may predominantly affect their ability to combat pathogens and metabolize harmful substances. Nevertheless, specific changes or effects require further investigation.

## 5 Conclusion

This study explored the differences in gut microbiota between Tibetan pigs and Yorkshire pigs using multi-omics approaches. It was found that under the “free-range + supplementary feeding” model, Tibetan pigs exhibited enhanced capabilities in short-chain fatty acid synthesis, as well as digestion of cellulose and hemicellulose. Moreover, unique gut genera such as *Rhodococcus* prevalent in high-altitude environments, contributed to Tibetan Pigs degrade a variety of natural compounds or metabolize toxic substances. In addition, the higher abundance of probiotics in the intestinal tract of Tibetan pigs likely plays a crucial role in maintaining intestinal homeostasis. These probiotics contribute to this balance through their potent bactericidal abilities and their capacity to metabolize toxic substances.

## Data Availability

The data presented in the study are deposited in the NCBI repository, the GSA database accession is CRA020245.
